# Somatic mutations may contribute to asymmetry in neurodegenerative disorders

**DOI:** 10.1093/braincomms/fcac184

**Published:** 2022-07-18

**Authors:** Christos Proukakis

**Affiliations:** Professor of Neurology and Neurogenetics, Department of Clinical and Movement Neurosciences, UCL Queen Square Institute of Neurology, London, UK

Left–right asymmetry in neurodegenerative disorders is widely recognized and is indeed a supportive criterion for idiopathic Parkinson’s disease in the Queen Square Brain Bank criteria. A number of plausible, and not mutually exclusive, explanations were recently suggested.^1^ The authors stated, however, that ‘the two brain hemispheres are genetically identical, at least in terms of DNA sequences’. This statement neglects the possibility that mosaicism due to somatic mutations, acquired post-zygotically, may be relevant to the question, if asymmetrically distributed, or indeed restricted to one hemisphere.

The existence of various types of somatic mutations in the brain, from single nucleotide variants (SNVs) to a wide range of structural variants including aneuploidy, has been well documented in recent years, with the help of emerging techniques such as single-cell whole-genome amplification.^2,3^ All types of somatic mutations may arise in development, with SNVs developing in early neurogenesis at a rate of ∼5 per cell per day.^4^ It is therefore not surprising that somatic mutations have a clear role in several neurodevelopmental disorders, including some with striking asymmetry such as hemimegalencephaly and focal cortical dysplasias, where relevant somatic mutations have been detected in surgically resected tissue.^3^ It is reasonable to speculate that these mutations would be absent or present at very low levels in the unaffected hemisphere, although this cannot be confirmed as healthy contralateral tissue is obviously not resected. Clear examples of somatic mutations arising in a lateralized fashion in development would, however, be informative. A landmark study of somatic SNVs from multiple human tissues showed marked left–right asymmetry.^5^ Although the brain was not included, these arose before gastrulation, suggesting that brain would also show this pattern. This has been now fully confirmed by detection of somatic SNVs by deep whole-genome sequencing of 25 brain regions from neurotypical individuals, which allowed the evaluation of the distribution of neural progenitor clones in neocortical development.^6^ Clones restricted to the brain were generally limited to one hemisphere, suggesting that in brain development separation along the midline occurs before an antero-posterior axis is established in each hemisphere. Inter-hemispheric asymmetry in mosaicism for somatic LINE-1 retrotransposon insertions has been demonstrated in the brain of a schizophrenia patient, with two insertions found to differ widely in levels between the same region across the two hemispheres.^7^

A role of somatic mutations in neurodegeneration is suggested by several lines of evidence.^8^ To support the hypothesis that they contribute to asymmetry, it would be important to demonstrate such examples in post-mortem disease tissue, but this is problematic for two reasons. In a given neurodegenerative disease, there may be preferential loss of cells with mutations which render them vulnerable, leading to spurious allelic fraction comparisons. From a practical point of view, in many cases brain banks only freeze one half of the brain, making such comparisons essentially impossible. Studies of *SNCA* CNVs in synucleinopathies^9,10^ and a report of two semantic dementia patients with somatic *TARDP* mutations and asymmetric temporal lobe atrophy^11^ only had access to a single frozen brain hemisphere, and therefore could not address this.

The possibility of somatic mutations contributing to left–right asymmetry of neurodegeneration needs to be considered, and robust investigation will only be possible when adequate whole frozen brains are available for detailed single-cell DNA sequencing studies.

## Data availability

Data sharing is not applicable to this article as no new data were created or analysed.

## Competing interests

The author reports no competing interests.

## References

[1] Lubben N, Ensink E, Coetzee GA, Labrie V. The enigma and implications of brain hemispheric asymmetry in neurodegenerative diseases. Brain Commun. 2021;3(3):fcab211. 10.1093/braincomms/fcab211 34557668 PMC8454206

[2] Rohrback S, Siddoway B, Liu CS, Chun J. Genomic mosaicism in the developing and adult brain. Dev Neurobiol. 2018;78(11):1026–1048. 10.1002/dneu.22626 30027562 PMC6214721

[3] Bizzotto S, Walsh CA. Genetic mosaicism in the human brain: From lineage tracing to neuropsychiatric disorders. Nat Rev Neurosci. 2022;23(5):275–286. 10.1038/s41583-022-00572-x 35322263

[4] Bae T, Tomasini L, Mariani J, et al Different mutational rates and mechanisms in human cells at pregastrulation and neurogenesis. Science. 2018;359(6375):550–555. 10.1126/science.aan8690 29217587 PMC6311130

[5] Park S, Mali NM, Kim R, et al Clonal dynamics in early human embryogenesis inferred from somatic mutation. Nature. 2021;597(7876):393–397. 10.1038/s41586-021-03786-8 34433967

[6] Breuss MW, Yang X, Schlachetzki JCM, et al Somatic mosaicism reveals clonal distributions of neocortical development. Nature. 2022;604(7907):689–696. 10.1038/s41586-022-04602-7 35444276 PMC9436791

[7] Zhu X, Zhou B, Pattni R, et al Machine learning reveals bilateral distribution of somatic L1 insertions in human neurons and glia. Nat Neurosci. 2021;24(2):186–196. 10.1038/s41593-020-00767-4 33432196 PMC8806165

[8] Proukakis C . Somatic mutations in neurodegeneration: An update. Neurobiol Dis. 2020;144:105021. 10.1016/j.nbd.2020.105021 32712267

[9] Perez-Rodriguez D, Kalyva M, Leija-Salazar M, et al Investigation of somatic CNVs in brains of synucleinopathy cases using targeted SNCA analysis and single cell sequencing. Acta Neuropathol Commun. 2019;7(1):219. 10.1186/s40478-019-0873-5 31870437 PMC6929293

[10] Mokretar K, Pease D, Taanman J-W, et al Somatic copy number gains of α-synuclein (SNCA) in Parkinson’s disease and multiple system atrophy brains. Brain. 2018;141(8):2419–2431. 10.1093/brain/awy157 29917054

[11] van Rooij J, Mol MO, Melhem S, et al Somatic TARDBP variants as a cause of semantic dementia. Brain. 2020;143(12):3827–3841. 10.1093/brain/awaa317 33155043 PMC7805802

